# Daily systemic energy expenditure in the acute phase of aneurysmal subarachnoid hemorrhage

**DOI:** 10.1080/03009734.2019.1659888

**Published:** 2019-12-17

**Authors:** Christoffer Nyberg, Elisabeth Ronne Engström, Lars Hillered, Torbjörn Karlsson

**Affiliations:** a Department of Neuroscience, Section of Neurosurgery, Uppsala University, Uppsala, Sweden;; b Department of Surgical Sciences, Section of Anesthesiology and Intensive Care, Uppsala University, Uppsala, Sweden

**Keywords:** Energy expenditure, indirect calorimetry, subarachnoid hemorrhage

## Abstract

**Background:** Patients with subarachnoid hemorrhage often have impaired consciousness and cannot regulate nutritional intakes themselves. Previous studies have demonstrated elevated energy expenditure in the acute phase, but it is not known whether the energy demand is constant during the first week after onset of the disease. In this study, we performed daily measurements of energy expenditure with indirect calorimetry during the first 7 days after aneurysmal subarachnoid hemorrhage in mechanically ventilated patients.

**Methods:** Metabolic measurements were performed daily with indirect calorimetry in 26 patients with aneurysmal subarachnoid hemorrhage. All patients were intubated and mechanically ventilated. The measured value was compared to the predicted values from the Harris–Benedict equation and the Penn State University 1998 equation. Urinary nitrogen excretion was measured daily.

**Results:** There was a significant increase in energy expenditure during days 2–3 compared to days 5–6. The Harris–Benedict equation underestimated metabolic demand. The Penn State 1998 equation was closer to the measured values, but still underestimated caloric need. Urinary nitrogen excretion increased throughout the first week from initially low values.

**Conclusions:** There is a dynamic course in energy expenditure in patients with aneurysmal subarachnoid hemorrhage, with increasing metabolic demand during the first week of the disease. Indirect calorimetry could be used more often to help provide an adequate amount of energy.

## Introduction

Spontaneous subarachnoid hemorrhage (SAH) is a potentially life-threatening condition that activates a stress response. This in turn triggers numerous events that prepares the body to meet the challenges of severe illness, including activation of the hypothalamic-pituitary-adrenal axis and of the sympathetic nervous system (1,2). The stress response also may influence the patient’s metabolic demand.

Apart from the initial impact at the time of onset of disease, there are several severe complications (e.g. vasospasm, rebleeding, hydrocephalus, and infections) that may affect energy expenditure (EE) in the acute phase. In critically ill patients, who cannot regulate nutritional intake themselves, nutritional treatment is especially important. It has previously been shown that the nutritional balance may have impact on the outcome after stroke (3,4). Underfeeding as well as overfeeding may affect outcome.

Predictive equations to estimate the nutritional need have been developed and refined since the early 1900s. The original equation by Harris and Benedict (H-B) from 1919 is still being used to calculate the basal metabolic rate (5). Several attempts have been made to make more accurate predictive equations, taking into account different factors that may influence energy balance. A patient with severe illness, sedation, and a need for mechanical ventilation presents an even more complex challenge. Numerous ways to predict EE in this heterogeneous group have been suggested. The equation from Penn State 1998 (PSU-98) (6) has previously been shown to correlate with measurements by indirect calorimetry (IC) for patients with severe traumatic brain injury (7). In this equation, H-B is combined with body temperature and respiratory minute volume to compensate for increased metabolism caused by the systemic inflammatory response.

IC is currently considered to be the most precise and widely available method for measuring EE in critically ill patients (8). However, the equipment is expensive, and in most centers IC is not routinely used. Measurements with IC on patients with hemorrhagic stroke have been reported. In most of these studies, single short measurements have been performed once or twice during the acute stage of the disease (9–14). In patients with SAH, measured EE has generally then been elevated compared to the energy demand predicted by equations.

The aim of this study was to investigate EE during the first week in patients with aneurysmal SAH. We carried out daily measurements with indirect calorimetry during the first week after onset of SAH to investigate if EE had a dynamic course. Daily urinary nitrogen excretion was measured to estimate protein catabolism. We also performed comparisons between measured and calculated EE using predictive equations.

## Materials and methods

### Patients and SAH management

Patients were included from October 2010 until July 2014. Mechanically ventilated patients with spontaneous SAH were considered for inclusion. Twenty-six patients were included with at least two measurements using indirect calorimetry during the first week after SAH. The patients were managed according to the Department of Neurosurgery’s protocols for SAH (15). Briefly, this includes early diagnosis and treatment of aneurysm. Patients with impaired consciousness were intubated and normo-ventilated. A ventricular catheter was placed for drainage of cerebrospinal fluid and monitoring of intracranial pressure. Sedation in mechanically ventilated patients was obtained, mainly with propofol and bolus doses of morphine. Enteral nutrition was administered through a gastro-enteral tube. Intravenous nutrition was only used in selected cases. Nimodipine was administered for three weeks. Symptoms of delayed cerebral ischemia were considered to be due to vasospasm when other causes of deterioration were ruled out. Vasospasm treatment was then given with increase in blood volume and blood pressure and in selected cases intra-arterial administration of nimodipine. Secondary insults putting further strain on the brain’s supply and use of energy and oxygen were systematically detected and treated if present (16). The Uppsala University Regional Ethical Review Board for clinical research granted ethical permission.

### Predictive equations

Calculations of the metabolic rate (*R*; kcal/day) were done for each patient with the Harris–Benedict equation, the Penn State 1998 equation, and by using 25 kcal per kilogram body weight.

(1) H-B men:R=66.473+(13.7516×W)+(5.0033×L)−(6.755×A)

(2) H-B women:R=655.0955+(9.5634×W)+(1.8496×L)−(4.6756×A)

(3) $$\text{Penn State}\;1998:\;R=\left(1.1\times \text{Harris}–\text{Benedict}\right)+\left(140\times T_{max}\right)+\left(32\times \dot{V}E\right)–5.340$$

where *W* is weight in kg, *L* is height in cm, *A* is age in years, *T_max_* is maximum temperature in °C in the past 24 hours, and *V̇E* is minute volume in L/min.

### Metabolic measurements

The energy expenditure was determined by measurements with the indirect calorimeter Quark RMR (COSMED, Rome, Italy). Before each measurement, the equipment was calibrated according to instructions from the manufacturer. The measurements lasted for approximately 4.5 hours. Records without V̇O_2_ and V̇CO_2_ values were removed. In order to exclude invalid measurements, values were removed that deviated more than two standard deviations from the mean. From the resulting data, daily energy expenditure was calculated by taking the median from all collected data points.

Measurements were started as soon as possible after admission, typically on the day after admission, and continued daily throughout the first week after SAH, or until the patient was no longer mechanically ventilated or had respiratory problems requiring >50% FiO_2_. On some occasions, patients were unavailable for measurements because of time-consuming examinations or surgical procedures.

In order to compare metabolic measurements between patients, the ratio of the measured value to H-B was calculated. Ratios of the measured values were also calculated to the PSU-98 estimates of EE, and to body weight. To be able to compare early and late measurements despite the fact that some patients were missing measurements, the mean of days 2–3 and the mean of days 5–6 were calculated for all patients.

Urine was collected for 24 hours repeatedly and analyzed for urea by the routine method used in the hospital chemistry department. From this value, daily urinary nitrogen excretion was calculated.

### Statistics

Statistical analyses were performed with R (R Foundation for Statistical Computing, Vienna, Austria). Changes in EE and nitrogen excretion over the entire time period were evaluated with the Skillings–Mack test. Wilcoxon matched pairs test was used to compare early and late measurements in days 2–3 and days 5–6. Values of *P* < 0.05 were considered statistically significant.

## Results

The mean age of the included patients was 59 years (range 41–81). Eighteen patients were of female gender. World Federation of Neurosurgical Societies SAH grade (17) at admission was I in 1 patient, II in 5 patients, III in 1 patient, IV in 15 patients, and V in 4 patients. All patients were diagnosed as having a ruptured aneurysm. The aneurysms were located in the anterior circulation in 21 patients, and in the posterior circulation in 5 patients. The aneurysms were treated with endovascular methods in 22 patients and with open surgery in 4 patients. Fourteen patients were conscious at discharge from the neurosurgical unit, 9 patients were unconscious, and 3 patients died before discharge.

**Figure 1.**
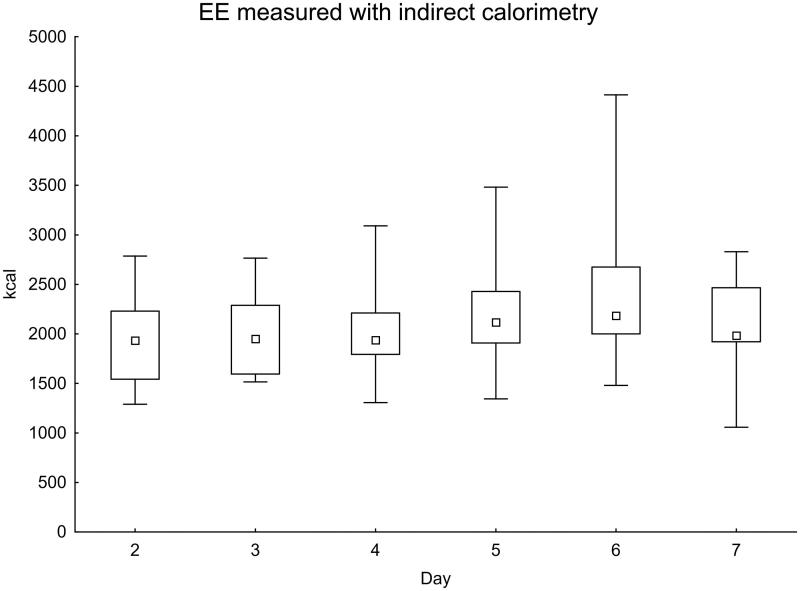
Daily EE measured with indirect calorimetry. Significance testing over time with the Skillings–Mack test, *P* = 0.030. Median with 25%–75% interquartile range and minimum–maximum values.

In total, 118 IC measurements were performed during the first week after onset of SAH. The measured values increased in the later part of the first week (Figure 1). When comparing days 2–3 with days 5–6, the difference was statistically significant (*P* = 0.017).

### Measured values versus predictive equations

**Figure 2.**
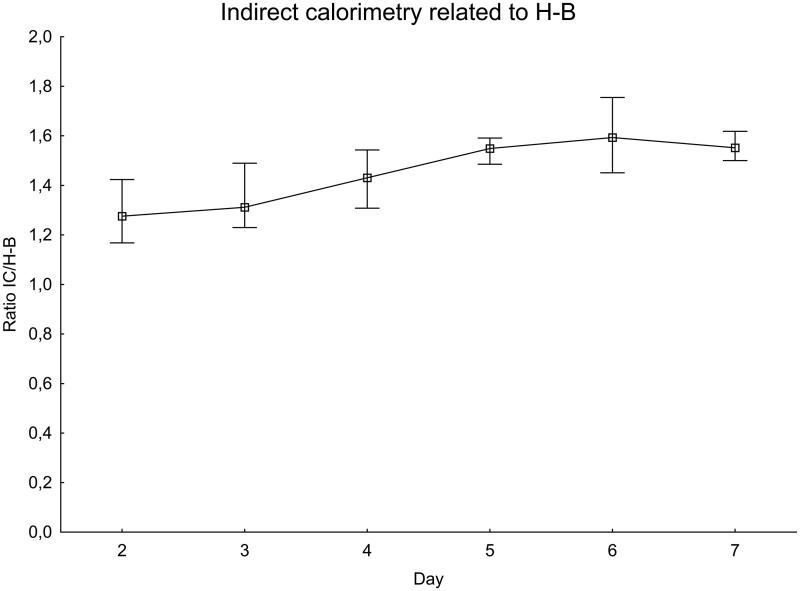
EE measured with indirect calorimetry related to the Harris–Benedict equation. Significance testing over time with the Skillings–Mack test, *P* = 0.030. Median with 25%–75% interquartile range.

**Figure 3.**
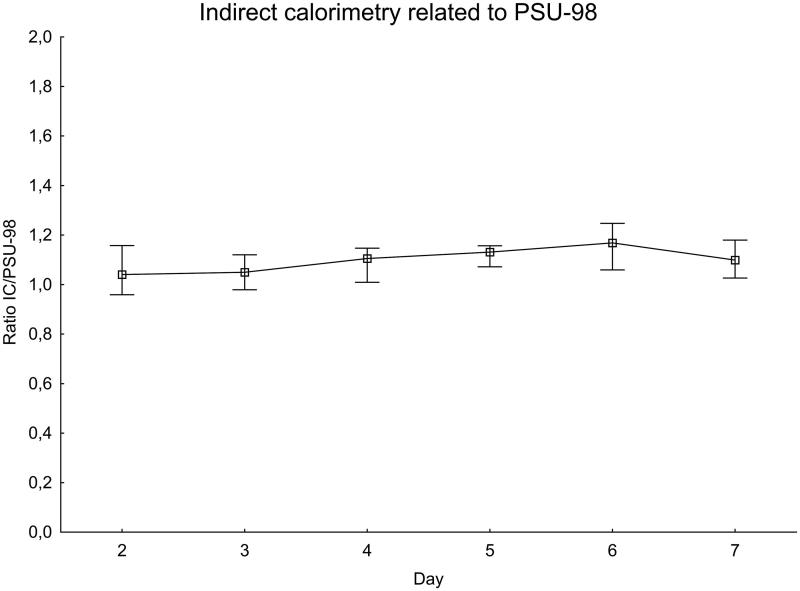
EE measured with indirect calorimetry related to the Penn State 1998 equation. Significance testing over time with the Skillings–Mack test, *P* = 0.12. Median with 25%–75% interquartile range.

For each patient and IC measurement, the ratio of IC to the expected value was calculated using the H-B equation (Figure 2) and the PSU-98 equation (Figure 3).

The ratio of IC to the H-B equation increased during the first week after SAH, peaking at day 6 when the measured value was about 60% higher than expected. There was a difference between days 2–3 and days 5–6, *P* = 0.020. The ratio of measured IC to the PSU-98 equation also increased in a similar manner. The variations were, however, smaller than with the H-B equation. On the day with the peak value, day 6, the measured value was less than 20% higher than expected.

**Figure 4.**
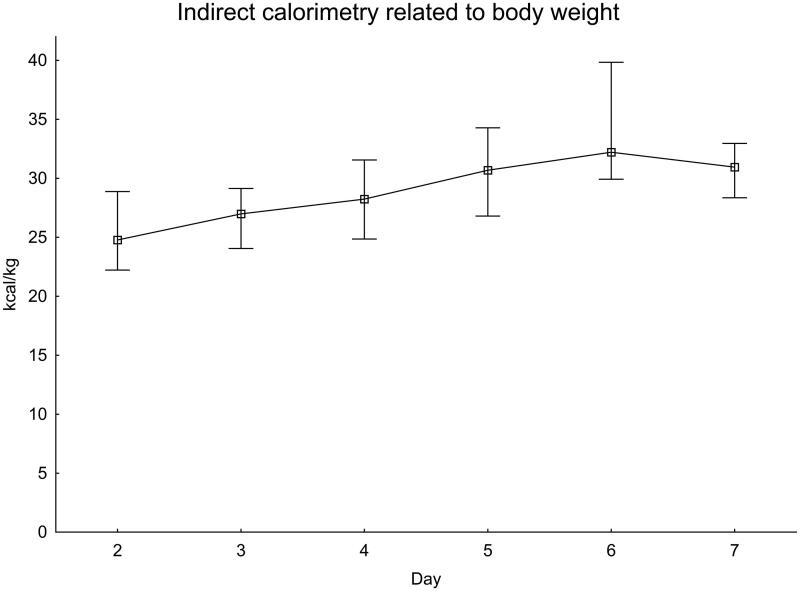
EE measured with indirect calorimetry related to body weight. Significance testing over time with the Skillings–Mack test, *P* = 0.041. Median with 25%–75% interquartile range.

The EE as measured with IC was also calculated to kg of body weight (Figure 4). EE was close to 25 kcal/kg in the first measurements and increased to more than 30 kcal/kg on day 6.

### Urinary nitrogen excretion

**Figure 5.**
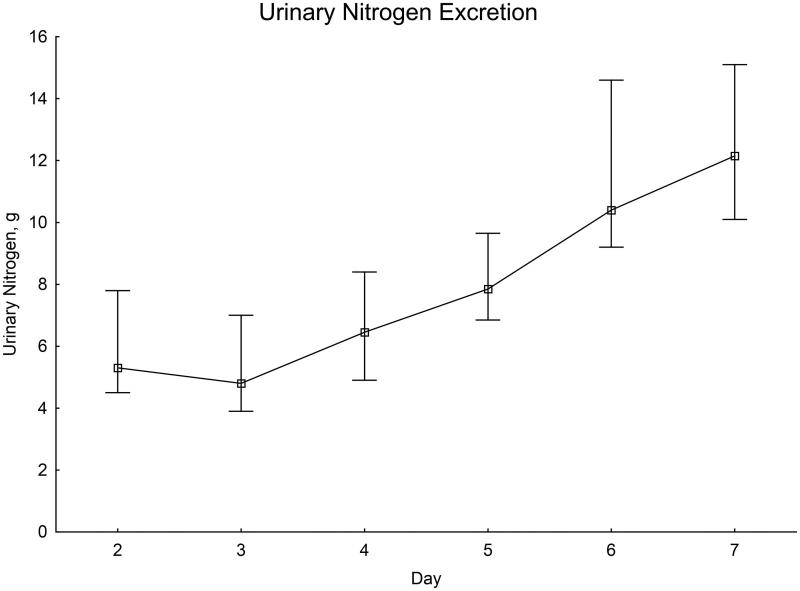
Daily nitrogen excretion in urine. Significance testing over time with the Skillings–Mack test, *P* < 0.001. Median with 25%–75% interquartile range.

From initially low values on the second and third day after SAH, there was a distinct increase throughout the first week (Figure 5).

## Discussion

Many patients with acute aneurysmal hemorrhage have impaired consciousness and cannot regulate their nutritional intake. Ensuring these patients an adequate caloric intake may be one factor affecting the outcome. To better understand their caloric requirements, we performed a study with repeated measurements using IC during the first week after SAH. To our knowledge, this is the first study with this high number of patients and daily repeated measurements with IC during the first 7 days after SAH.

One difficulty when comparing EE between and within patients is the fact that the basal metabolism varies with multiple factors such as age, body weight, height, gender, and systemic inflammatory state. In order to be able to compare measured EE in this group of patients, we used the ratio of measured EE to the H-B equation. The predicted EE with the H-B equation is constant during the first week, since it is based on constant or slowly changing factors. This means that the calculated ratio can be used not only to compare EE within patients during the first week after SAH, but also between patients. This calculation can also be used to compare EE measured with IC to the expected value from the H-B equation. From our results, it seems clear that the H-B equation underestimates the metabolic demand in this patient group. Since the H-B equation was developed for prediction of the basal metabolic rate in healthy individuals, it is not surprising that the equation does not apply without modification on patients with severe illness.

We performed the same type of calculation with another predictive method, the PSU-98 equation. This is one of the equations developed to predict caloric need in an intensive care unit (ICU) setting. It is based on the H-B equation, but also includes information about respiration and body temperature to compensate for the increased metabolism caused by the systemic inflammatory response. Our results indicate that the PSU-98 equation may underestimate EE in patients with SAH, although the difference between predicted and calculated EE is smaller than when the H-B equation is used. Also, the increase in EE that we found during the course of the disease was accounted for by the PSU-98 equation since no significant difference was found. This suggests that the PSU-98 equation is a better alternative than the H-B equation for this patient group. The variation between patients is still substantial, and the perfect predictive equation for SAH patients is yet to be found.

One way of deciding how much energy to provide to a patient in intensive care is to estimate 25 kcal per kg body weight (18). This calculation is most likely widely used because of its simplicity. With the patients in this study, using the 25 kcal/kg equation underestimates EE, especially in the later part of the first week. On day 6, measured EE was above 30 kcal/kg.

Urinary nitrogen excretion can be used as an indicator of protein turnover. Our measurements showed low nitrogen excretion the first days after onset of disease. Thereafter, there seemed to be a linear increase in nitrogen excretion. A similar pattern has been demonstrated in plasma measurements in previous studies (19). One possible interpretation is a shift in protein metabolism towards catabolism. Whether the possible catabolic state is only due to the disease itself is unclear. The increase in EE during the acute phase after SAH is almost parallel to nitrogen excretion, raising the suspicion that the proteolytic state could be enhanced by underfeeding. In this study, we did not collect detailed information about nitrogen intake. This means we cannot rule out high protein intake as a factor influencing nitrogen excretion, but the measurements of EE or nitrogen excretion did not influence the type of nutrition or energy provided to the patients. One factor possibly contributing to the low values the first day is the fact that enteral nutrition in our unit is not provided the first day after onset of disease, thereby causing low protein metabolism.

Measured EE increased gradually during the first 6 days, and there was a significant difference in EE between days 2–3 compared to days 5–6, in line with recent investigations (14). An increase in metabolic demand could be expected after an intracranial aneurysm rupture and the subsequent triggering of a stress response. The findings in this study suggest that EE has a dynamic course after SAH, and it is difficult to rely on a predictive equation to determine nutritional requirements. In order to provide an adequate amount of energy to SAH patients, it would be beneficial to routinely use IC. Our results illustrate that a single measurement with IC is only valid on the day of the measurement and cannot be used universally during the first week after SAH.

There are some limitations of our study. The metabolic measurements with IC was only performed on mechanically ventilated patients, meaning that mostly poor-grade patients were included. Furthermore, the limited number of patients in the study made it impossible to compare between different groups of patients based on treatment modality, complications, and outcome.

We conclude that energy expenditure in mechanically ventilated patients with aneurysmal SAH increases gradually during the first week after SAH. Prediction of metabolic demand with equations is often unreliable. In this study, the PSU-98 equation performed best of those tested but, on the whole, underestimated EE. These results suggest that IC could be beneficial in patients with SAH and mechanical ventilation.
